# Guide to writing and publishing a scientific manuscript: Part 1—The structure

**DOI:** 10.1007/s43678-021-00241-5

**Published:** 2021-12-21

**Authors:** Ian G. Stiell, For the Canadian EM Writing Group, Paul Atkinson, Peter Cameron, Alix Carter, Warren Cheung, Ryan Chuang, Kerstin Wit, Quynh Doan, Ian Drennan, Debra Eagles, Andrew Hall, Ariel Hendin, Grant Innes, Eddy Lang, Patrick McLane, Andrew McRae, Catherine Patocka, Jeffrey J. Perry, Naveen Poonai, Venkatesh Thiruganasambandamoorthy, Daniel Ting, Christian Vaillancourt, Robert Woods, Krishan Yadav, Peter Zed

**Affiliations:** 1 Department of Emergency Medicine, University of Ottawa, Ottawa Hospital Research Institute, Ottawa, ON Canada; 2 Clinical Epidemiology Unit, F657, The Ottawa Hospital, 1053 Carling Avenue, Ottawa, ON K1Y 4E9 Canada

## Introduction

Writing a scientific manuscript for a peer-reviewed medical journal can be a frustrating but ultimately very satisfying process. Benefits for the authors include the ability to share the results of their project with a large audience and the opportunity to change practice, the satisfaction of completing a challenging scholarly endeavor, and the recognition of your institution in terms of advancement and/or compensation. Those who have not yet written a scientific manuscript may not appreciate how long and intensive the journey can be. The goal of this guide is to offer tried and true, step by step, recommendations on how to simplify the writing process and increase the chances of successful publication. Variations on these tips have been presented nationally and internationally and have been the basis for authoring or co-authoring hundreds of papers as well as for mentoring many learners in the Canadian emergency medicine community. Part 1 will address the structure or anatomy of a manuscript and Part 2 will look at the process of writing and dealing with journals.

## Specific sections

### Example manuscript

- See online appendix.

### Tables and figures

- Generally, these should be finalized before writing the Results and Discussion sections.
- These should be **presented sequentially** as cited in the text, first the Tables then Figures, at the end of the manuscript or in a separate document. See **online appendix**
  - *The typesetter will determine their actual position in the published paper.*
- **Titles** should be short but self-explanatory, such that the Tables and Figures stand alone without the reader having to go back to the text.
- For clinical journals, we suggest making the Tables and Figures **easy to read**:
  - round to **one decimal** where possible.
  - give only percentages without counts when there are **multiple columns**.
  - keep headings brief and use **footnotes** to explain terms and abbreviations.
- Avoid use of **p-values** except for the primary outcomes; focus on differences and CIs.
- When reporting on socio-demographic characteristics, use terms that are **inclusive, describe diversity,** and considerate of marginalized populations.
- Most journals **limit the number** of Tables and Figures such that some may have to be moved to the online appendix.
- **Figures or graphs** should be specifically prepared with simple lines for publication; do not use 3-D or PowerPoint slides.

### Title page

- **Title** should only give design if strong, e.g., randomized trial, systematic review, meta-analysis; avoid “retrospective” or “chart review” as these terms may devalue your paper.
  - *A few journals may insist on giving the design in the title*
- **Author Information**, names, affiliations.
- **Disclaimers,** e.g., findings represent the work of the authors and not their institution.
- **Sources of Support**, grants, other funding.
- **Word count**, text only excluding abstract and tables.
- **Keywords**, 3–5 commonly used terms.
- **Other sections** may be placed elsewhere in the manuscript:
  - **Disclosures** of conflicts and relationships, usually given on the ICJME form.
  - **Acknowledgements** of non-authors: research staff, clinicians.
  - **Author contributions**.

### Abstract

- Adhere to the **structured format and word count** required by the specific journal.
  - Common subheadings are: Introduction, Methods, Results, Conclusion.
- Use only **standard abbreviations** in the abstract and main text; non-standard abbreviations can be confusing to readers and reviewers.
  - Standard abbreviations should be **introduced** at the first use.
  - Examples of standard abbreviations are ED, CPR, CT, ECG.
  - Examples of non-standard abbreviations to be avoided include: LOS, SRS, IND, CCLS, RCSA.
- ICMJE recommends placing **clinical trial registration number** at the end of the abstract.

### Introduction

- Should be brief and no more than **3 paragraphs**.
- Paragraph 1: definition, epidemiology, and importance of the **health-care problem**.
- Paragraph 2: key studies and **knowledge gap** to be addressed.
- Paragraph 3: **Goal and specific objectives** of the paper and how the gap will be addressed.
  - For **educational articles**, the approach should be grounded in or framed within educational theory or principles

### Methods

- Authors should be aware of and generally follow accepted **reporting guidelines**, based upon the study type whether clinical trial, systematic review, observational study, etc.
  - These are well presented on the **EQUATOR website** [1].
  - *A few journals require completion and submission of the relevant checklist.*
- Use standard **subheadings**: Study Design, Study Setting, Participants, Interventions (if any), Outcome Measures, Data Collection, Data Analysis and Sample Size.
  - Use “Research Questions” in **Qualitative research**.
- **“Health records review”** is preferable to “retrospective chart review.”
- Indicate **Research Ethics Board** approval or waiver, usually at end of Study Participants.
- **Educational research** often involves one of many different quantitative, qualitative, or synthesis research approaches.

### Results

- Generally, **one paragraph** for each Table and Figure.
- Aim for brevity by reporting only **important or interesting findings;** do not repeat everything from the Tables.
- In the text, give **percentages** and the number from which the percentage was calculated.
  - e.g., “12.1% of 410 participants…”
- Avoid **starting a sentence** with a number.
  - e.g., use “Of all subjects, 3.1% were…”; not “3.1% of subjects were….”
- We suggest using **words for spelling out numerals 1–10** (e.g., “three”) and numerals above that.

### Discussion

- This is the section that many authors **struggle with**; we strongly recommend using the paragraphs described below.
- We also encourage use of **subheadings** which will aid reviewers and readers, even though most journals do not require them.
  - All subheadings are usually **one paragraph maximum** except Previous Studies.
- **1. Interpretation.** Highlight the main findings without repeating numbers and present the take home messages.
- **2. Previous Studies.** Discuss how your findings compare to prior studies, trying to emphasize what is unique.
- **3. Strengths and Limitations.** Discuss methodological strengths and weaknesses, trying to mitigate limitations.
- **4. Clinical Implications.** Be explicit in how your findings can impact and improve care, either at the patient or the system level.
- **5. Research Implications.** Suggest future directions of enquiry and knowledge gaps.
- **6. Conclusion.** Highlight your unique findings in this important section; state explicitly how your study can improve care; align with Abstract conclusion.
  - Conclusions must not reach beyond the study findings.
  - Never, ever conclude by saying **“future research required”** as that immediately tells reviewers and readers that your findings are not meaningful.
- **Educational papers** typically link interpretation and the existing literature together (rather than as separate paragraphs).

### References

- Always use **reference software** such as EndNote, Mendeley, or others.
- Most medical journals use the **Vancouver style**, citing references with Arabic numbers in the text and providing a list at the end [2].

## Supplementary Information

Below is the link to the electronic supplementary material.

Supplementary file1 (DOCX 130 KB)

Supplementary file2 (DOCX 67 KB)
