# Supplementary material for: Guide to writing and publishing a scientific manuscript: Part 1—The structure
Source: CJEM. 2021 Dec 21;24(2):117–9. doi: 10.1007/s43678-021-00241-5 (PMC8904337; doi:10.1007/s43678-021-00241-5)
Supplement: Supplementary file 1 — Supplementary file1 (DOCX 130 KB) [file 43678_2021_241_MOESM1_ESM.docx]

Adverse events associated with electrical cardioversion in patients

with acute atrial fibrillation and atrial flutter

**Ian G. Stiell, MD, MSc**

**Debra Eagles, MD, MSc**

**Marie-Joe Nemnom, MSc**

**Erica Brown, BSc**

**Monica Taljaard, PhD**

**For the RAFF Investigators**

**Mailing Address: Ian G. Stiell, MD, MSc**

**Clinical Epidemiology Unit, F657, The Ottawa Hospital**

**1053 Carling Avenue**

**Ottawa, Ontario, Canada K1Y 4E9**

**Telephone: 613-798-5555 ext 18683 Fax: 613-761-5351**

**E-mail:** [**istiell@ohri.ca**](mailto:istiell@ohri.ca) **Twitter: @EMO_Daddy**

**OCRID ID: 0000-0002-2583-6408**

**Word Count: 2,542**

**Date: 26 July 2021**

***RAFF Investigators:***

**Ian G. Stiell, MD, MSc**

**Debra Eagles, MD, MSc**

**Marie-Joe Nemnom, MSc**

**Erica Brown, BSc**

**Monica Taljaard, PhD**

**Patrick M. Archambault, MD, MSc**

**David Birnie, MD**

**Bjug Borgundvaag, MD, PhD**

**Gregory Clark, MD**

**Philip Davis, MD, MSc**

**Danny Godin, MD**

**Corinne Hohl, MD, MHSc**

**Bernard Mathieu, MD**

**Andrew D. McRae, MD, PhD**

**Eric Mercier, MD, MSc**

**Judy Morris, MD, MSc**

**Ratika Parkash, MD, MSc**

**Jeffrey J. Perry, MD, MSc**

**Brian H. Rowe, MD, MSc**

**Venkatesh Thiruganasambandamoorthy, MBBS, MSc**

**Frank Scheuermeyer, MD, MHSc**

**Marco L.A. Sivilotti, MD, MSc**

**Alain Vadeboncoeur, MD**

**Department of Emergency Medicine, University of Ottawa; Ottawa Hospital Research Institute, Ottawa, Ontario (IS, DE, JJP, VT);**

**Clinical Epidemiology Program, Ottawa Hospital Research Institute, University of Ottawa, Ottawa, Ontario (EB, MJN);**

**Clinical Epidemiology Program, Ottawa Hospital Research Institute, School of Epidemiology and Public Health, University of Ottawa, Ottawa, Ontario (MT);**

**Division of Emergency Medicine, University of Toronto, Schwartz/Reisman Emergency Medicine Institute, Mount Sinai Hospital, Toronto, Ontario (BB);**

**Department of Emergency Medicine, University of Saskatchewan, Saskatoon, Saskatchewan (PD);**

**Department of Emergency Medicine, McGill University Health Centre, Montreal, Québec (GC);**

**Division of Cardiology, Department of Medicine, Dalhousie University, Dartmouth, Nova Scotia (RP)**

**Département de médecine de famille et de médecine d’urgence, Université de Montréal, Montreal, Québec (JM, BM)**

**Department of Emergency Medicine, University of British Columbia, Vancouver, British Columbia (FS)**

**Institut de Cardiologie de Montréal, Université de Montréal,** **Montreal, Québec (AV);**

**Department of Emergency Medicine, Centre for Clinical Epidemiology and Evaluation,**

**Département de médecine familiale et de médecine d’urgence, Centre de Recherche du CHU de Québec, Université Laval, Quebec City, Québec (EM);**

**Département de médecine familiale et de médicine d’urgence et Département d'anesthésiologie et de soins intensifs, Centre de recherche du Centre intégré de santé et de services sociaux de Chaudière-Appalaches, Université Laval, Quebec City, Québec (PA);**

**Department of Emergency Medicine, Queen’s University, Kingston, Ontario (MLAS);**

**Division of Cardiology, University of Ottawa Heart Institute, University of Ottawa, Ottawa, Ontario (DB);**

**Institut de Cardiologie de Montréal, Université de Montréal, Montréal, Québec (AV;**

**Department of Emergency Medicine, Centre for Clinical Epidemiology and Evaluation, Vancouver Coastal Health Research Institute, University of British Columbia, Vancouver, British Columbia (CMH);**

**Department of Emergency Medicine, and Community Health Sciences, University of Calgary, Calgary, Alberta (ADM);**

**Department of Emergency Medicine, and School of Public Health, University of Alberta, Edmonton, Alberta (BHR).**

***Funding and Conflicts***

The study was funded by the Department of Emergency Medicine, University of Ottawa, Ottawa, Canada; and by an unrestricted grant of less than $20,000 from InCarda Therapeutics Inc, Newark, California, USA. The latter had no impact on the design, analysis, or data analysis nor the manuscript.

The authors report no conflicts of interest.

***Acknowledgements***

We thank our staff Angela Marcantonio, Carolyne Kennedy, and Maddie Crabtree.

# ABSTRACT

**BACKGROUND:**

We sought to evaluate safety of electrical cardioversion (ECV) for patients with acute atrial fibrillation (AF) or atrial flutter (AFL) in the emergency department (ED).

**METHODS:**

This was an analysis of data from four multicentre AF/AFL studies conducted between 2008 and 2019 at 23 large EDs. We included adult patients who received attempts at ECV and who had presented acutely after symptom onset. Staff manually reviewed study and clinical records to abstract data.

**RESULTS:**

We evaluated 1,736 ECV cases with mean age 60.1 years and male 67.1%. The overall success of ECV was 90.2% (95% CI 88.7-91.6%) with 4.9% of patients admitted. ED physicians performed the ECV in 95.2% and provided sedation in 96.5%. 13.9% (12.3-15.7%) of cases experienced important adverse events that required treatment and 0.4% were classified life-threatening. Another 5.6% had adverse events that did not require treatment. Logistic regression found that the RAFF3 study cohort (OR 2.0), age ≥85 years (OR 2.1), coronary artery disease (OR 1.5), midazolam (OR 1.9), and fentanyl (OR 1.5) were associated with important adverse events.

**CONCLUSIONS:**

This large evaluation of the safety of ECV for acute AF/AFL in the ED found that while serious adverse events were rare, there were a concerning number of events following sedation that required intervention. Physicians should be aware that older age, coronary artery disease, and fentanyl are associated with a higher risk of important adverse events. This study provides more information for shared decision-making discussions with patients when choosing between drug-shock and shock-only cardioversion strategies.

**Word count: 246**

**Key Words:** Emergency Department, Atrial Fibrillation, Cardioversion, Patient Safety

# INTRODUCTION

Acute atrial fibrillation (AF) and flutter (AFL) are the most common arrhythmias requiring management in the emergency department (ED). AF is characterized by disorganized micro-reentrant atrial electrical activity leading to an irregular and rapid ventricular response. Acute AFL is less common and is characterized by a macro-reentrant circuit within the atria leading to more regular atrial depolarizations.^1-3^ Our focus in the ED is on episodes of acute AF or AFL which are usually less than 48 hours in duration and are highly symptomatic, requiring rapid treatment.^4^ We estimate that there are 500,000 acute AF and AFL visits annually to the ED in Canada and the U.S.^5 6^

Overall, Canadian ED physicians favor an early rhythm control approach and national guidelines support this.^4 7^ There is equipoise between physicians preferring to start with pharmacological cardioversion and then proceeding to electrical cardioversion (ECV) if necessary (drug-shock strategy) and those who prefer to start with ECV (shock-only strategy). Both approaches have been shown to be effective in restoring normal sinus rhythm and allowing early discharge home from the ED. Canadian ED physicians generally provide their own sedation and conduct the ECVs themselves rather than involving consultants. While the effectiveness and safety of pharmacological cardioversion has been well studied, little research has been done on the safety of ECV in the ED for patients with acute AF/AFL.

 The overall goal of this study was to evaluate the safety of ECV for acute AF/AFL patients in the ED. We did not include elective cardioversions, which are rarely performed in the ED. Specifically, this study evaluated adverse events associated with sedation and ECV and considered them as serious, requiring treatment, and not requiring treatment. We also evaluated the success rate of ECV.

# METHODS

### Design

This was a secondary analysis of existing data sets of four previous multicentre AF/AFL studies conducted by the Principal Investigator.^8-12^ These studies had a total enrollment of 3,475 patients. RAFF0 was a health records review of all acute AF/AFL patients seen at eight EDs over 12 months (N=1,068; enrolled 2008).^8^ RAFF1 was a prospective cohort study of all acute AF/AFL patients seen at six hospitals over 2 years (N=1,091; enrolled 2010-2012).^9^ RAFF2 was a randomized controlled trial that enrolled consenting acute AF/AFL patients (N=472; enrolled 2013-2018).^10 11^ RAFF3 was a stepped-wedge cluster randomized trial that enrolled all acute AF/AFL patients seen at 11 EDs (N=844; enrolled 2018-2019).^12^ Use of cardioversion strategies was at the discretion of the treating physician except in RAFF2 where it was driven by the randomization process.

*Setting*

Twenty-three different Canadian academic and community hospital EDs in seven provinces participated in one or more of the four previous multicentre studies.

### Participants

The four original studies had similar inclusion criteria which was stable patients treated at least three hours and not more than seven days after onset of acute AF or AFL, where symptoms prompted ED management by rhythm or rate control. We excluded patients who had any of a) permanent or persistent AF; b) deemed unstable and required immediate ECV; c) primary presentation was for another condition rather than arrhythmia, e.g. pneumonia, pulmonary embolism, and sepsis, etc. For this analysis, we only included patients who underwent attempted ECV. We originally obtained local Research Ethics Board approvals for all patients in the original studies, as well as approval from the Ottawa Health Science Network Research Ethics Board for this analysis.

### Outcome Measures

The outcome measures were determined *a priori* in the study protocol. The primary outcome measure was *serious adverse event* occurring prior to discharge from the ED and which was defined by a consensus of the investigators as:

a. Hypotension (systolic blood pressure < 90 mmHg) with signs of clinical instability, requiring vasopressor or inotropic agent;

b. Bradycardia with signs of clinical instability (e.g. hypotension) requiring pacing or chronotropic agent;

c. Sinus pause post-conversion, requiring pacing or CPR;

d. Ventricular arrhythmia – sustained ventricular tachycardia ≥ 30 seconds, torsade de pointes, ventricular fibrillation, or cardiac arrest [and whether due to lack of synchronization];

e. AFL with 1:1 AV conduction requiring intervention (DC cardioversion) for termination, or an IV AV nodal blocking agent (beta‑blocker or calcium-channel blocker) to slow the ventricular rate; or

f. Any arrhythmia with signs of clinical instability (e.g. hypotension) and requires DC cardioversion, defibrillation, pacing, or vasopressor, inotropic or chronotropic agent.

The secondary outcome measure was *other adverse event requiring treatment*:

a. Hypotension: systolic blood pressure < 90 mm-Hg requiring a fluid bolus of minimum 250 mL;

b. Respiratory events: hypoxia (O2 saturation < 90%), aspiration, or airway maneuvers (e.g., jaw positioning, oral airway, bag-valve-mask ventilation, and intubation);

c. Prolonged time to recover from conscious sedation (≥ 30 min);

d. Gastrointestinal side effects (nausea and vomiting);

e. Post-sedation agitation requiring treatment; or

f. Any other adverse events requiring treatment, e.g. administration of atropine

We considered the combination of serious and other adverse events requiring treatment to be *“important adverse events*.”

We also identified *other adverse events not requiring treatment*:

a. Conduction problems: development of new bundle branch block or QTc lengthening to > 480 msec or > 20 msec above baseline;

b. Other dysrhythmias: bradycardia (heart rate < 50 bpm), sinus pauses post-conversion (any duration);

c. Skin burns;

d. Patient complaint of pain from cardioversion; or

e. Post-sedation agitation not requiring treatment.

Finally, we defined successful cardioversion as return to normal sinus rhythm immediately after all ECV shocks administered and persisting for at least 30 minutes.

### Data Sources

We reviewed the electronic datasets of the four prior studies and then our trained staff manually reviewed the case record forms as well as original ED nursing and physician notes of the patients who underwent electrical cardioversion. They abstracted additional information regarding adverse events associated with ECV and then entered it into a new electronic database. The physician investigators reviewed any uncertainties brought forward by the research staff and resolved these by consensus. The two older studies had paper-based case record forms which were retrieved from storage. The four datasets were then merged for analysis.

### Sample Size and Data Analysis

We conducted no a priori sample size calculation but of 3,475 total patients in the four studies, we expected to include 1,745 who underwent attempts at ECV. We conducted simple descriptive analyses and reported frequencies with percentages and 95% confidence intervals (CI) for categorical variables and means with standard deviations (SD) or medians with interquartile ranges (IQR) for continuous variables, as appropriate. Subgroup analyses, specified *a priori*, were performed for rhythm (AF vs AFL), those who received an antiarrhythmic agent prior to ECV (yes vs no), and duration of symptoms (> or ≤48 hours). To identify factors associated with important adverse events we used multivariable logistic regression with the visit as the unit of analysis. The dependent variable was the presence of any important adverse event.

Independent variables were selected based on clinical relevance and included an indicator for the parent study, as well as sex, age, CHADS_2_ criteria, other medical history, initial ECG rhythm, antiarrhythmic medication given in the ED, rate control agent given in ED, total shocks given, sedation given, and fentanyl use. Coronary artery disease was defined as history of any of myocardial infarction, bypass surgery, PCI, or taking medication for angina. All variables were considered for the model. Continuous variables were categorized at clinically meaningful cutpoints. Multicollinearity was ruled out using variance inflation factors (VIF). Visits with missing data were excluded from this analysis. Goodness of fit was examined using Hosmer-Lemeshow test and C-statistic. We conducted all analyses using Statistical Analysis Software (SAS) version 9.4.

# RESULTS

Of 3,475 available patient visits from January 1, 2008 to September 30, 2019, 1,736 cases underwent attempted ECV (Figure 1). While nine case records could not be located, no included patients were lost to follow-up for the primary outcome.

The RAFF2 Study contributed the lowest proportion of cases (17.6%) (Table 1). The included patients had a mean age of 60.1 years, although 3.5% were age 85 years or greater. On arrival to the ED, 83.9% of patients were in AF and 16.1% in AFL. The mean duration of symptoms was 13.9 hours, 73.1% had prior episodes of acute AF/AFL, and 51.3% had a CHADS_2_ score of one or more.

Overall, 776 (44.6%) patients received anti-arrhythmic drugs, most commonly intravenous procainamide (Table 2). ECV (95.2%) and sedation (96.5%) were almost always provided by an emergency physician. The overall success of ECV was 90.2% (95% CI 88.7-91.6%) and only 4.9% of patients were admitted.

Overall, 18.0% of cases experienced an adverse event and only seven (0.4%) were classified as serious cardiac adverse events (Table 3, Supp Table S1). There were no deaths or strokes in the ED. There were 240 (13.8%) adverse events requiring treatment, with the most common being hypotension and respiratory events. Of these were 159 (9.2%) cases that required airway manoeuvres such as jaw positioning, ventilation, or an airway. The overall incidence of important adverse events was 13.9% (95% CI 12.3-15.7%). Another 98 cases (5.6%) had adverse events that did not require treatment.

Table 4 shows several factors that are different between patients with and without important adverse events. The logistic regression analysis (Table 5) identified five variables independently associated with important adverse events: RAFF3 study cohort compared to RAFF0 (OR 2.0; 95% CI 1.3-2.9), age ≥85 years (OR 2.1; 95% CI 1.1-4.0), a history of coronary artery disease (OR 1.5; 95% CI 1.0-2.3), use of midazolam (OR 1.9; 95% CI 1.0-3.4), and use of fentanyl with the procedural sedation (OR 1.5; 95% CI 1.1-2.1). Of note, neither use of a rhythm control agent (OR 1.1; 95% CI 0.8-1.5) nor a rate control agent (OR 1.1; 95% CI 0.8-1.5) in the ED were associated with adverse events.

We found very little difference when comparing patients who received anti-arrhythmic drugs in the ED versus those who did not, between cases of AF and AFL, and whose onset was less than or greater than 48 hours (Supp Tables S2, S3, S4).

# DISCUSSION

### Interpretation

This review of cases from four large multicentre AF/AFL studies is the largest reported evaluation of the safety of ECV in the ED. Most procedures were conducted by ED physicians without the presence of consultant and there was an overall 90% success in restoring sinus rhythm. While serious adverse events were uncommon, there were a surprising proportion of cases with adverse events requiring intervention as well as a smaller proportion that did not require treatment. Factors associated with important adverse events were the RAFF3 study cohort, age ≥85 years, history of coronary disease, use of midazolam compared to propofol, and use of fentanyl with the procedural sedation. These results suggest that physicians should be cautious providing ECV to older patients and judicious in their use of midazolam and fentanyl. This information should also assist in shared decision-making discussions with patients when choosing between drug-shock and shock-only strategies for AF/AFL in the ED.

### Previous Studies

Relatively few studies have evaluated the safety and effectiveness of ECV for AF/AFL in the ED, most were conducted retrospectively, and all devised their own list of adverse events.^13-15^ In a prospective study, Scheuermeyer and colleagues noted adverse events in 24.2% of 62 patients undergoing ECV with none being major.^16^ Butler and colleagues found that 714 patients undergoing procedural sedation suffered hypotension (27.6%) and respiratory issues (26.6%) but did not report arrhythmias or success rate.^17^ Bonfanti reported 96.2% success amongst 419 cases and only 9 skin burns but did not comment on use of anti-arrhythmic drugs.^18^ Fried described a success rate of 88% amongst 887 cases with very few receiving aa drugs, and a 14% rate of complications.^19^ In contrast, our pooled study of 1,736 cases found ECV success rate of 90.1.% with 44.7% of patients having received prior anti-arrhythmic medication in the ED. We identified a larger number of both cardiac and respiratory adverse events than prior studies.

Our observation that fentanyl was associated with more frequent adverse events is consistent with the expected properties of an opioid, as previously reported when procedural sedation is performed for a variety of conditions beyond ECV. Two randomized clinical trials in emergency patients found fentanyl and the closely related opioid alfentanil caused more respiratory adverse events when added to propofol for procedural sedation.^20 21^ The emergency literature is less clear regarding a safety signal for midazolam versus propofol, with a systematic review having found mostly observational cohort studies, and that inconsistent definition of adverse events in four trials precluded meta-analysis.^15^ A more recent randomized trial found that midazolam caused clinically relevant oxygen desaturation, although propofol was more likely to result in brief apnea.^22^

### Limitations

Patient selection in the RAFF2 Trial was limited to patients providing written consent. In all studies, we included patients chosen for ECV by the attending physician. These issues could have introduced selection bias, but we think this is unlikely due to the very large number of hospitals and physicians participating. While RAFF0 did not use a prospective data collection process, the other studies did. To ensure consistency of adverse event coding, our highly trained staff manually reviewed the clinical notes of every included patient. We did not adhere to standard patient safety methodology because we assessed the presence or absence of pre-defined adverse events which required little interpretation or agreement.^23 24^ We recognize that the skill of ED physicians in providing procedural sedation has improved since 2008 and that midazolam is rarely used at present. We are uncertain why the RAFF3 cohort had a higher proportion of important adverse events but possible factors could be that this is a chance finding, that there was a slightly different patient population, or that the 11 EDs in this study had relatively little prior experience with ECV.

### Clinical and Research Implications

### While current Canadian AF/AFL guidelines encourage rhythm control in the ED for acute AF/AFL, the choice between electrical or pharmacological cardioversion is left to the physician and patient preference.^7 25-30^ Nevertheless, we are aware that many Canadian physicians prefer to use ECV initially rather than a drug-shock approach which starts with an anti-arrhythmic drug and proceeds to ECV if necessary. Both approaches are highly effective as shown in the RAFF2 Trial.^10^ The current study, however, identifies a worrisome number of issues associated with ECV, particularly due to the sedation required. Canadian ED physicians are trained to provide sedation for a variety of procedures and are well versed in the use of fast-acting agents like propofol. Nevertheless, physicians should be aware that older age, coronary artery disease, and addition of fentanyl to procedural sedation put patients at higher risk of adverse events. Overall, we recommend that the choice between drug-shock and shock-only strategies should be a shared decision between the patient and the physician.

This study has clearly documented the frequency and severity of adverse events associated with ECV of acute AF/AFL in the ED. Future research might focus on the impact of best practice guidelines on reducing the occurrence of such events in the ED.

### Conclusions

This is the largest reported evaluation of the efficacy and safety of ECV for acute AF/AFL in the ED and found overall 90% success. While serious adverse events were rare, there were a surprising proportion of cases with adverse events requiring intervention as well as a smaller proportion that did not require treatment. These data should assist physicians understand that older age, coronary artery disease, and addition of fentanyl are associated with higher risk of important adverse events during sedation and ECV. This study provides more information for shared decision-making discussions with patients when choosing between drug-shock and shock-only strategies for acute AF/AFL in the ED.

# REFERENCES

1. Kirchhof P, Benussi S, Kotecha D, et al. 2016 ESC Guidelines for the management of atrial fibrillation developed in collaboration with EACTS. *Eur Heart J* 2016;37(38):2893-962.

2. Scheuermeyer FX, Grafstein E, Heilbron B, et al. Emergency department management and 1-year outcomes of patients with atrial flutter. *Ann Emerg Med* 2011;57(6):564-71.

3. Hamilton A, Clark D, Gray A, et al. The epidemiology and management of recent-onset atrial fibrillation and flutter presenting to the Emergency Department. *Eur J Emerg Med* 2015;22(3):155-61. doi: 10.1097/mej.0000000000000198 [published Online First: 2014/09/10]

4. Stiell IG, Macle L. Canadian cardiovascular society atrial fibrillation guidelines 2010: management of recent-onset atrial fibrillation and flutter in the emergency department. *Can J Cardiol* 2011;27(1):38-46.

5. Pitts SR, Niska RW, Xu J, et al. National Hospital Ambulatory Medical Care Survey: 2006 emergency department summary. *Natl Health Stat Report* 2008(7):1-38.

6. Chan TB, Schull MJ, Schultz SE. Emergency department services in Ontario 1993-2000. Toronto, Ontario: Institute for Clinical Evaluative Sciences 2001.

7. Stiell IG, Scheuermeyer FX, Vadeboncoeur A, et al. CAEP Acute Atrial Fibrillation/Flutter Best Practices Checklist. *Can J Emerg Med* 2018;20(3):334-42.

8. Stiell IG, Clement CM, Brison RJ, et al. Variation in management of recent-onset atrial fibrillation and flutter among academic hospital emergency departments. *Ann Emerg Med* 2011;57(1):13-21.

9. Stiell IG, Clement CM, Rowe BH, et al. Outcomes for ED Patients with Recent-onset Atrial Fibrillation and Flutter (RAFF) Treated in Canadian Hospitals. *Ann Emerg Med* 2017;69(5):562-71.

10. Stiell IG, Sivilotti MLA, Taljaard M, et al. Electrical versus pharmacological cardioversion for emergency department patients with acute atrial fibrillation (RAFF2): a partial factorial randomised trial. *Lancet* 2020;395(10221):339-49. doi: 10.1016/s0140-6736(19)32994-0 [published Online First: 2020/02/03]

11. Stiell IG, Sivilotti MLA, Taljaard M, et al. A randomized, controlled comparison of electrical versus pharmacological cardioversion for emergency department patients with acute atrial flutter. *Cjem* 2021;23(3):314-24. doi: 10.1007/s43678-020-00067-7 [published Online First: 2021/05/08]

12. Stiell IGA, P.M.; Morris, J.; Mercier, E.; Eagles, D.; Perry, J.J.; Scheuermeyer, F.; Clark, G.; Gosselin, S.; Vadeboncoeur, A.; Parkash, R.; de Wit, K.; Patey, A.; Thiruganasambandamoorthy, V.; Taljaard, M.; For the RAFF3 Study Investigators. RAFF-3 Trial: A Stepped-Wedge Cluster Randomized Trial to Improve Care of Acute Atrial Fibrillation and Flutter in the Emergency Department. *Can J Cardiol* 2021;Accepted

13. Domanovits H, Schillinger M, Thoennissen J, et al. Termination of recent-onset atrial fibrillation/flutter in the emergency department: a sequential approach with intravenous ibutilide and external electrical cardioversion. *Resuscitation* 2000;45:181-87.

14. Dankner R, Shahar A, Novikov I, et al. Treatment of stable atrial fibrillation in the emergency department: a population-based comparison of electrical direct-current versus pharmacological cardioversion or conservative management. *Cardiology* 2009;112(4):270-8. doi: 10.1159/000151703 [published Online First: 2008/09/26]

15. Hohl CM, Sadatsafavi M, Nosyk B, et al. Safety and clinical effectiveness of midazolam versus propofol for procedural sedation in the emergency department: a systematic review. *Acad Emerg Med* 2008;15(1):1-8.

16. Scheuermeyer FX, Andolfatto G, Christenson J, et al. A mulitcenter randomized trial to evaluate a chemical-first or electrical-first cardioversion strategy for patients with uncomplicated acute atrial fibrillation. *Acad Emerg Med* 2019;26:969-81.

17. Butler M, Froese P, Zed P, et al. Emergency department procedural sedation for primary electrical cardioversion - a comparison with procedural sedations for other reasons. *World J Emerg Med* 2017;8(3):165-69. doi: 10.5847/wjem.j.1920-8642.2017.03.001 [published Online First: 2017/07/07]

18. Bonfanti L, Annovi A, Sanchis-Gomar F, et al. Effectiveness and safety of electrical cardioversion for acute-onset atrial fibrillation in the emergency department: a real-world 10-year single center experience. *Clin Exp Emerg Med* 2019;6(1):64-69. doi: 10.15441/ceem.17.286 [published Online First: 2019/04/05]

19. Fried AM, Strout TD, Perron AD. Electrical cardioversion for atrial fibrillation in the emergency department: A large single-center experience. *Am J Emerg Med* 2021;42:115-20. doi: 10.1016/j.ajem.2020.02.001 [published Online First: 2020/02/26]

20. Messenger DW, Murray HE, Dungey PE, et al. Subdissociative-dose ketamine versus fentanyl for analgesia during propofol procedural sedation: a randomized clinical trial. *Acad Emerg Med* 2008;15(10):877-86. doi: 10.1111/j.1553-2712.2008.00219.x [published Online First: 2008/08/30]

21. Miner JR, Gray RO, Stephens D, et al. Randomized clinical trial of propofol with and without alfentanil for deep procedural sedation in the emergency department. *Acad Emerg Med* 2009;16(9):825-34. doi: 10.1111/j.1553-2712.2009.00487.x [published Online First: 2009/10/23]

22. Lameijer H, Sikkema YT, Pol A, et al. Propofol versus midazolam for procedural sedation in the emergency department: A study on efficacy and safety. *Am J Emerg Med* 2017;35(5):692-96. doi: 10.1016/j.ajem.2016.12.075 [published Online First: 2017/01/12]

23. Network APS. Adverse Events, Near Misses, and Errors 2019 [updated September 2019. Available from: <https://psnet.ahrq.gov/primer/adverse-events-near-misses-and-errors> accessed May 2021.

24. Calder L, Forster A, Nelson M, et al. Adverse events among patients registered in high acuity areas of the emergency department: a prospective cohort study. *Canadian Journal of Emergency Medicine* 2010;12(5):421-30.

25. Andrade JG, Verma A, Mitchell LB, et al. 2018 Focused Update of the Canadian Cardiovascular Society Guidelines for the Management of Atrial Fibrillation. *Can J Cardiol* 2018;34(11):1371-92.

26. Stiell IG, McMurtry MS, McRae A, et al. The Canadian Cardiovascular Society 2018 guideline update for atrial fibrillation - A different perspective. *CJEM* 2019;21(5):572-75. doi: 10.1017/cem.2019.399 [published Online First: 2019/09/26]

27. Stiell IG, McMurtry MS, McRae A, et al. Safe Cardioversion for Patients With Acute-Onset Atrial Fibrillation and Flutter: Practical Concerns and Considerations. *Can J Cardiol* 2019;35(10):1296-300. doi: 10.1016/j.cjca.2019.06.007 [published Online First: 2019/09/10]

28. Andrade JG, Mitchell LB. Periprocedural Anticoagulation for Cardioversion of Acute Onset Atrial Fibrillation and Flutter: Evidence Base for Current Guidelines. *Can J Cardiol* 2019;35(10):1301-10. doi: 10.1016/j.cjca.2019.06.006.

29. Andrade JG, Aguilar M, Atzema C, et al. The 2020 Canadian Cardiovascular Society/Canadian Heart Rhythm Society Comprehensive Guidelines for the Management of Atrial Fibrillation. *Can J Cardiol* 2020;36(12):1847-948. doi: 10.1016/j.cjca.2020.09.001 [published Online First: 2020/11/17]

30. Stiell IG, de Wit K, Scheuermeyer FX, et al. 2021 CAEP Acute Atrial Fibrillation/Flutter Best Practices Checklist. *Cjem* 2021 doi: 10.1007/s43678-021-00167-y [published Online First: 2021/08/13]
