# Supplementary material for: Guide to writing and publishing a scientific manuscript: Part 1—The structure
Source: CJEM. 2021 Dec 21;24(2):117–9. doi: 10.1007/s43678-021-00241-5 (PMC8904337; doi:10.1007/s43678-021-00241-5)
Supplement: Supplementary file 2 — Supplementary file2 (DOCX 67 KB) [file 43678_2021_241_MOESM2_ESM.docx]

# Table 1. Characteristics of 1,736 Visits of Patients with Acute Atrial Fibrillation or Flutter Who Received Electrical Cardioversion

| **Characteristic** | **Visits  N=1,736** |
| --- | --- |
| **Age in Years, Mean (SD)** | 60.1 (15.1) |
| Range | 17 – 97 |
| **Male (%)** | 1,164 (67.1) |
| **Study (%)** |  |
| RAFF-0 | 421 (24.3) |
| RAFF-1 | 567 (32.7) |
| RAFF-2 | 305 (17.6) |
| RAFF-3 | 443 (25.5) |
| **Province (%)** |  |
| Ontario | 694 (40.0) |
| Alberta | 458 (26.4) |
| Quebec | 258 (14.9) |
| Saskatchewan | 133 (7.7) |
| Nova Scotia | 102 (5.9) |
| British Columbia | 48 (2.8) |
| New Brunswick | 43 (2.5) |
| **Duration of Arrhythmia, Mean (SD)** |  |
| Hours (48 Hours or Less) | 1,645 (94.8) |
| Range | 1 – 48 |
| Days (Between 3 And 7^#^ Days) | 91 (5.2) |
| Range | 3 – 10 |
| **Main Presenting Symptom (%)** |  |
| Palpitations | 1,392 (80.2) |
| Chest Pain | 182 (10.5) |
| Shortness of Breath | 59 (3.4) |
| Dizziness | 46 (2.6) |
| Weakness | 24 (1.4) |
| Syncope | 7 (0.4) |
| None | 1 (0.1) |
| Other | 25 (1.4) |
| **Initial Vital Signs, Mean (SD)** |  |
| Heart Rate, bpm | 119.3 (30.2) |
| Systolic Blood Pressure, mmHg | 131.7 (22.2) |
| Oxygen Saturation, % | 97.5 (2.0) |
| On Room Air (%) | 1,562 (90.0) |
| **CTAS* Level, Median (IQR) (n=1,730)** | 2 (2 – 3) |
| **Previous Atrial Fibrillation (%)** | 1,269 (73.1) |
| Electrical Cardioversion | 807 (46.5) |
| Pharmacologic Cardioversion | 312 (18.0) |
| Ablation | 185 (10.7) |
| **CHADS_2_ Criteria (%)** |  |
| Age ≥ 75 Years | 308 (17.7) |
| Stroke / TIA | 84 (4.8) |
| Hypertension | 710 (40.9) |
| Diabetes Mellitus | 143 (8.2) |
| Congestive Heart Failure | 51 (2.9) |
| **CHADS_2_ Score^^^, Median (IQR)** | 1 (0 – 1) |
| Score ≥ 1 (%) | 891 (51.3) |
| **Other Medical History (%)** |  |
| Coronary Artery Disease | 210 (12.1) |
| Valvular Heart Disease | 139 (8.0) |
| Pacemaker/ICD | 41 (2.4) |
| COPD/Asthma | 141 (8.1) |
| **Medications at Presentation (%)** |  |
| Beta Blocker | 639 (36.8) |
| Antiplatelet (n=1,315) | 345 (26.2) |
| Warfarin | 324 (18.7) |
| Calcium Channel Blocker | 262 (15.1) |
| Antiarrhythmic | 371 (21.4) |
| Direct Oral Anticoagulant (n=1,315) | 350 (26.6) |
| LMW Heparin (n=872) | 4 (0.5) |
| **Investigations (%)** |  |
| Initial ECG Rhythm |  |
| Atrial Fibrillation | 1,456 (83.9) |
| Atrial Flutter | 280 (16.1) |

COPD=chronic obstructive pulmonary disease; ECG=electrocardiogram; ICD=internal cardiac defibrillator; IQR=InterQuartile Range; LMW= low molecular weight; SD=Standard Deviation; TIA=transient ischemic attack.

^#^One patient had an onset of >7days

*Canadian Triage and Acuity Scale, ranges from 1 (critical) to 5 (not urgent)

^^^The CHADS_2_ score ranges from 0 to 6

# Table 2. Treatments Given in ED and Disposition for 1,736 Visits of Patients with Atrial Fibrillation or Flutter

| **Treatment (%)** | **Visits**  **N=1,736** |
| --- | --- |
| **Antiarrhythmics given in ED (N=774)** | **774 (44.6)** |
| Procainamide IV | 638 (82.4) |
| Amiodarone IV | 66 (8.5) |
| Adenosine IV | 43 (5.6) |
| Propafenone PO | 18 (2.3) |
| Sotalol PO | 12 (1.6) |
| Flecainide PO | 7 (0.9) |
| Ibutilide IV | 5 (0.6) |
| Dronedarone PO | 1 (0.1) |
| Vernakalant IV | 1 (0.1) |
| **Rate Control Given in ED (N=516)** | **516 (29.7)** |
| Metoprolol | 336 (65.1) |
| Diltiazem | 158 (30.6) |
| Other Beta Blocker^+^ | 9 (1.7) |
| Digoxin | 7 (1.4) |
| Verapamil | 2 (0.4) |
| **Electrical Cardioversion in ED (N=1,736)** | **1,736 (100)** |
| Overall Success (%) | 1,566 (90.2) |
| First Shock Successful (%) | 1,345 (77.5) |
| Total Shocks Given, Median (IQR) (n=1,730) | 1 (1 – 1) |
| ≥ 3 shocks (%) | 156 (9.0) |
| Initial Energy Used (%) (n=1,659) |  |
| < 200 Joules | 774 (46.7) |
| 200 Joules | 843 (50.8) |
| > 200 Joules | 42 (2.5) |
| Pad Position (%) (n=525) |  |
| Antero-lateral | 205 (39.0) |
| Antero-posterior | 320 (61.0) |
| Physician Doing Cardioversion (%) (n=1,734) |  |
| Emergency Physician | 1,651 (95.2) |
| Cardiologist | 83 (4.8) |
| Sedation Given by (%) (n=1,731) |  |
| Emergency Physician | 1,671 (96.5) |
| Anesthesiologist | 60 (3.5) |
| Sedation Given* (%) |  |
| Propofol | 1,642 (94.6) |
| Fentanyl | 921 (53.1) |
| Ketamine | 123 (7.1) |
| Midazolam | 77 (4.4) |
| Etomidate | 18 (1.0) |
| Methohexital | 5 (0.3) |
| **Disposition (%)** |  |
| Discharged | 1,651 (95.1) |
| Admitted | 85 (4.9) |
| **In sinus rhythm at disposition (%)** | 1,633 (94.1) |
| **Converted by (%) (n=1,633)** |  |
| Electrical | 1,566 (95.9) |
| Drug | 37 (2.3) |
| Spontaneous | 30 (1.8) |
| **Return Visit within 30 days (%) (n=1,314) **** | **213 (16.2)** |
| Stroke | 1 (0.1) |
| Death^^^ | 1 (0.1) |

^+^Atenolol, Bisoprolol, Labetalol

*Some patients received more than one agent

^^^Death due to metastatic rectal cancer

** Missing for some cases

# Table 3. Adverse Events Occurring from Electrical Cardioversion and/or Procedural Sedation

| **Adverse Events (%)** | **Visits**  **N=1,736** |
| --- | --- |
| **Adverse Events*** | **313 (18.0)** |
| **Important Adverse Events*** | **242 (13.9)** |
| **Serious Cardiac Adverse Events*** | **7 (0.4)** |
| Hypotension (SBP <90) Requiring Vasopressors or Inotropes | 3 (0.2) |
| Sinus Pause | 3 (0.2) |
| Bradycardia | 2 (0.1) |
| Ventricular Arrhythmia | 1 (0.1) |
| Atrial Flutter | 0 (0.0) |
| Any Arrhythmia with Clinical Instability | 0 (0.0) |
| **Other Adverse Event Requiring Treatment*** | **240 (13.8)** |
| Hypotension (SBP <90) Requiring IV Fluid Bolus | 60 (3.5) |
| Respiratory Events | 179 (10.3) |
| Airway Maneuvers | 159 (9.2) |
| Jaw Positioning | 116 (6.7) |
| Bag-Valve-Mask Ventilation | 32 (1.8) |
| Oral Airway | 2 (0.1) |
| Hypoxia (O_2_ Saturation <90%) | 57 (3.3) |
| Aspiration | 3 (0.2) |
| Requiring Narcan | 1 (0.1) |
| Prolonged Time to Recover (≥30 Min) | 2 (0.1) |
| Gastrointestinal Side Effects | 15 (0.9) |
| Post-Sedation Agitation Requiring Treatment | 1 (0.1) |
| Other | 4 (0.2) |
| **Adverse Event Not Requiring Treatment*** | **98 (5.6)** |
| Conduction Problems | 19 (1.1) |
| Other Dysrhythmias | 54 (3.1) |
| Skin Burns | 5 (0.3) |
| Pain | 13 (0.7) |
| Post-Sedation Agitation Not Requiring Treatment | 3 (0.2) |
| Other | 8 (0.5) |

SBP=systolic blood pressure

*Patients may have had more than one adverse event

# Table 4. Characteristics of Visits with Important Adverse Events versus No Important Adverse Events

|  | **Important Adverse Event** | **No Important Adverse Event** |
| --- | --- | --- |
|  | **N=242 (13.9%)** | **N=1,494 (86.1%)** |
| **Study (%)** |  |  |
| RAFF-0 | 54 (22.3) | 367 (24.6) |
| RAFF-1 | 54 (22.3) | 513 (34.3) |
| RAFF-2 | 42 (17.4) | 263 (17.6) |
| RAFF-3 | 92 (38.0) | 351 (23.5) |
| **Age in Years, Mean (SD)** | **62.7 (15.5)** | **59.7 (15)** |
| Range | 17 – 93 | 18 – 97 |
| **Male (%)** | **157 (64.9)** | **1007 (67.4)** |
| **CHADS2 Criteria (%)** |  |  |
| Age ≥ 75 years | 55 (22.7) | 253 (16.9) |
| Congestive Heart Failure | 6 (2.5) | 45 (3.0) |
| Hypertension | 112 (46.3) | 598 (40.0) |
| Diabetes Mellitus | 18 (7.4) | 125 (8.4) |
| Stroke / TIA | 13 (5.4) | 71 (4.8) |
| CHADS2 Score, Median (IQR) | 1 (0 – 1) | 1 (0 – 1) |
| CHADS65 Positive (%) | 164 (67.8) | 910 (60.9) |
| **Other Medical History (%)** |  |  |
| Coronary Artery Disease | 41 (16.9) | 169 (11.3) |
| Pacemaker / ICD | 7 (2.9) | 34 (2.3) |
| COPD / Asthma | 23 (9.5) | 118 (7.9) |
| **Medications at Presentation (%)** |  |  |
| Antiarrhythmic | 59 (24.4) | 312 (20.9) |
| Direct Oral Anticoagulant (n=188:1,127) | 66 (35.1) | 284 (25.2) |
| **Initial ECG rhythm (%)** |  |  |
| Atrial fibrillation | 197 (81.4) | 1,259 (84.3) |
| Atrial flutter | 45 (18.6) | 235 (15.7) |
| **Medications Given in ED (%)** |  |  |
| Antiarrhythmic | 113 (46.7) | 661 (44.2) |
| Rate Control | 80 (42.6) | 436 (38.7) |
| **Total shocks given (%) (n=241:1,489)** |  |  |
| < 3 | 213 (88.4) | 1,361 (91.4) |
| ≥ 3 | 28 (11.6) | 128 (8.6) |
| **Sedation Used (%) (n=241:1,480)** |  |  |
| Propofol | 224 (92.6) | 1,418 (94.9) |
| Ketamine | 17 (7.0) | 106 (7.1) |
| Midazolam | 19 (7.9) | 58 (3.9) |
| Etomidate | 5 (2.1) | 13 (0.9) |
| **Fentanyl Used (%) (n=241:1,480)** | 145 (60.2) | 776 (52.4) |
| **Disposition (%)** |  |  |
| Discharged | 224 (92.6) | 1427 (95.5) |
| Admitted | 18 (7.4) | 67 (4.5) |

# Table 5. Factors Independently Associated with Important Adverse Events as Determined by Multivariable Logistic Regression

| **N=1,716** | **OR (95% CI)** | **p-value** |
| --- | --- | --- |
| **Study** |  | **<0.001** |
| RAFF-0 | ref |  |
| RAFF-1 | 0.73 (0.48; 1.10) |  |
| RAFF-2 | 1.25 (0.75; 2.08) |  |
| RAFF-3 | **1.96 (1.33; 2.90)** |  |
| **Male** | 1.01 (0.74; 1.37) | 0.97 |
| **Age ≥ 85 years** | **2.12 (1.11; 4.04)** | **0.02** |
| **CHADS_2_ criteria** |  |  |
| Congestive Heart Failure | 0.77 (0.32; 1.87) | 0.56 |
| Hypertension | 1.14 (0.84; 1.53) | 0.40 |
| Diabetes Mellitus | 0.66 (0.38; 1.14) | 0.14 |
| Stroke / TIA | 1.05 (0.56; 1.97) | 0.88 |
| **Other Medical History** |  |  |
| Coronary Artery Disease | **1.53 (1.03; 2.28)** | **0.04** |
| Pacemaker / ICD | 1.13 (0.48; 2.67) | 0.78 |
| COPD / Asthma | 1.18 (0.72; 1.93) | 0.51 |
| **Initial ECG rhythm** |  | 0.14 |
| Atrial fibrillation | ref |  |
| Atrial flutter | 1.32 (0.91; 1.91) |  |
| **Medications Given in ED** |  |  |
| Antiarrhythmic | 1.07 (0.77; 1.49) | 0.68 |
| Rate Control | 1.09 (0.80; 1.49) | 0.60 |
| **Total shocks given** |  | 0.07 |
| < 3 | ref |  |
| ≥ 3 | 1.52 (0.96; 2.39) |  |
| **Sedation Given** |  |  |
| Propofol | 1.01 (0.47; 2.19) | 0.98 |
| Ketamine | 0.98 (0.53; 1.80) | 0.94 |
| Midazolam | **1.86 (1.02; 3.41)** | **0.04** |
| Etomidate | 1.76 (0.47; 6.65) | 0.40 |
| **Fentanyl Used** | **1.53 (1.13; 2.07)** | **0.006** |

Hosmer-Lemeshow test: df=8; p-value=0.81

C-statistic (95%CI): 0.66 (0.62; 0.69)

# Figure 1. Inclusion of Cases from the Four Study Cohorts

**Eligible for Enrolment: 1,745**

RAFF0: 426

RAFF1: 571

RAFF2: 305

RAFF3: 443

**Original Study: 3,475**

RAFF0: 1,068

RAFF1: 1,091

RAFF2: 472

RAFF3: 844

**Not Eligible for Enrolment: 1,730**

RAFF0: 642

RAFF1: 520

RAFF2: 167

RAFF3: 401

*Not Electrically Cardioverted*

*Electrically Cardioverted*

**Included: 1,736**

RAFF0: 421

RAFF1: 567

RAFF2: 305

RAFF3: 443

**Missing (could not locate): 9**

RAFF0: 5

RAFF1: 4

RAFF2: 0

RAFF3: 0

# Supplementary Table S1. Seven Cases with Serious Adverse Events following Electrical Cardioversion

| **Age/Sex (Study)** | **Disposition** | **Survived** | **Summary of SAE** |
| --- | --- | --- | --- |
| 69/M (RAFF-0) | Discharged | Yes | This patient experienced an episode of hypotension (71/54 mmHg) and bradycardia (HR 45 bpm) post ECV. They were treated with normal saline, Atropine, Narcan and Ephedrine. |
| 60/M (RAFF-0) | Discharged | Yes | This patient experienced a sinus pause of approximately 25 seconds post ECV. They were treated with 1mg Atropine (no note of CPR). |
| 49/M (RAFF-2) | Admitted | Yes | This patient was inadvertently electrically cardioverted without synchronization, resulting in decompensated ventricular fibrillation. The patient was given CPR right away, and was shocked again with 360kJ without success. The patient was also treated with several rounds of Epinephrine, Amiodarone bolus, calcium bicarbonate, and several more shocks. Double defibrillator pads were added to chest walls and patient was shocked again with ROSC. Down time was a total of approximately 13 minutes. The patient’s blood pressure was low and treated with phenylephrine transiently. |
| 76/F (RAFF-3) | Discharged | Yes | This patient experienced a long sinus pause after electrically cardioversion. There is no supporting documentation of any treatment for this. |
| 32/M (RAFF-3) | Discharged | Yes | This patient experienced a syncopal event post ECV as he was recovering from the ketamine given. His heart rate decreased to the 40’s and his blood pressure became non-palpable. Chest compressions were briefly initiated. He was given atropine 0.5 mg IV after which he regained consciousness and his heart rate improved to a normal rate. |
| 68/M (RAFF-3) | Discharged | Yes | This patient experienced an apneic period for approximately 3 minutes which was treated with BVM. The patient also experienced a brief hypotensive episode (SBP 80 mmHg) which was successfully treated with normal saline and 100mcg of Phenylephrine. |
| 83/M (RAFF-3) | Admitted | Yes | This patient experienced 2 sinus pauses post ECV, each lasting approximately 4 seconds. The patient was not given any treatment or CPR. |

# Supplementary Table S2. Atrial fibrillation vs. Atrial flutter

| **Adverse Events (%)** | **Atrial fibrillation** | **Atrial flutter** |
| --- | --- | --- |
|  | **N=1,456 (83.9%)** | **N=280 (16.1%)** |
| **Successful Electrical Cardioversion in ED** | **1312 (90.1)** | **254 (90.7)** |
| **Adverse Events*** | **262 (18.0)** | **51 (18.2)** |
| **Important Adverse Event*** | **197 (13.5)** | **45 (16.1)** |
| **Serious Cardiac Adverse Events*** | **7 (0.5)** | **0 (0.0)** |
| Hypotension (SBP <90) Requiring Vasopressors or Inotropes | 3 (0.2) | 0 (0.0) |
| Sinus Pause | 3 (0.2) | 0 (0.0) |
| Bradycardia | 2 (0.1) | 0 (0.0) |
| Ventricular Arrhythmia | 1 (0.1) | 0 (0.0) |
| Atrial Flutter | 0 (0.0) | 0 (0.0) |
| Any Arrhythmia with Clinical Instability | 0 (0.0) | 0 (0.0) |
| **Other Adverse Event Requiring Treatment*** | **193 (13.3)** | **45 (16.1)** |
| Hypotension (SBP <90) Requiring IV Fluid Bolus | 51 (3.5) | 9 (3.2) |
| Respiratory Events | 139 (9.5) | 40 (14.3) |
| Airway Maneuvers | 121 (8.3) | 38 (13.6) |
| Jaw Positioning | 86 (5.9) | 30 (10.7) |
| Bag-Valve-Mask Ventilation | 27 (1.9) | 5 (1.8) |
| Oral Airway | 2 (0.1) | 0 (0.0) |
| Hypoxia (O_2_ Saturation <90%) | 47 (3.2) | 10 (3.6) |
| Aspiration | 2 (0.1) | 1 (0.4) |
| Requiring Narcan | 1 (0.1) | 0 (0.0) |
| Prolonged Time to Recover (≥30 Min) | 2 (0.1) | 0 (0.0) |
| Gastrointestinal Side Effects | 14 (1.0) | 1 (0.4) |
| Post-Sedation Agitation Requiring Treatment | 1 (0.1) | 0 (0.0) |
| Other | 4 (0.3) | 0 (0.0) |
| **Adverse Event Not Requiring Treatment*** | **87 (6.0)** | **11 (3.9)** |
| Conduction Problems | 16 (1.1) | 3 (1.1) |
| Other Dysrhythmias | 48 (3.3) | 6 (2.1) |
| Skin Burns | 5 (0.3) | 0 (0.0) |
| Pain | 12 (0.8) | 1 (0.4) |
| Post-Sedation Agitation Not Requiring Treatment | 3 (0.2) | 0 (0.0) |
| Other | 7 (0.5) | 1 (0.4) |

SBP=systolic blood pressure

*Patients may have had more than one adverse event
